# GLP-1 receptor agonists and risk of suicide or suicide attempts – A nationwide cohort and self-controlled case series study

**DOI:** 10.1038/s41380-026-03619-y

**Published:** 2026-04-18

**Authors:** Christoffer Stanislaus, Matilde Winther-Jensen, Sharleny Stanislaus, Merete Osler, Klara Coello, Maj Vinberg

**Affiliations:** 1 Section of Data, Biostatistics and Pharmacoepidemiology, Center for Clinical Research and Prevention, Bispebjerg and Frederiksberg Hospitals, Frederiksberg, Denmark; 2 Department of Clinical Medicine, Faculty of Health and Medical Sciences, University of Copenhagen, Copenhagen, Denmark; 3 The Early Multimodular Prevention and Intervention Research Institution (EMPIRI), Psychiatric Center, Copenhagen University Hospital – North Zealand, Copenhagen, Denmark; 4 Copenhagen Affective Disorders Research Centre (CADIC), Psychiatric Center Copenhagen, Frederiksberg, Denmark; 5 Section of Epidemiology, Department of Public Health, University of Copenhagen, Copenhagen, Denmark

**Keywords:** Neuroscience, Depression

## Abstract

The European Medicines Agency (EMA) and the U.S. Food and Drug Administration (FDA) have raised concerns about a potential link between glucagon-like peptide-1 receptor agonists (GLP-1 RAs) and suicide or suicide attempts. We conducted two new-user, active comparator cohort studies. The GLP1-RAs vs sodium-glucose cotransporter-2 (SGLT-2) inhibitors cohort included new users of GLP-1 RAs (*n* = *83,464*) or SGLT-2 inhibitors (*n* = *78,366*), and the GLP1-RAs vs dipeptidyl peptidase-4 (DPP-4) inhibitors cohort included new users of GLP-1 RAs (*n* = *108,322*) or DPP-4 inhibitors (*n* = *55,411*). We also employed a self-controlled case series design to compare suicide, or suicide attempts before and after GLP-1 RA treatment initiation across three time periods. In the cohort analyses patients who initiated GLP-1 RAs did not differ in the hazard ratio (HR) for suicide or suicide attempts from SGLT-2 inhibitor users (HR: 0.93; 95% CI: 0.57–1.52), and GLP-1 RA users had a lower risk of suicide or suicide attempts compared with DPP-4 inhibitor users (HR: 0.58; 95% CI: 0.37–0.91). In the self-controlled case series design, use of GLP-1 RAs was associated with a lower incidence rate ratio (IRR) of suicide or suicide attempts one year after treatment initiation (IRR: 0.45; 95% CI: 0.10–0.50) and 13–24 months after treatment initiation compared with pretreatment. This study showed that use of GLP-1 RAs was not associated with increased incidence of suicide or suicide attempts in either the active comparator new-user design or in the self-controlled case series design.

## Introduction

The Glucagon-like peptide-1 receptor agonist (GLP-1 RA) has been approved by the U.S. Food and Drug Administration (FDA) and the European Medicines Agency (EMA) to treat diabetes for more than a decade and has recently been authorized to treat overweight and obesity (FDA 2021, EMA 2022) [1]. GLP-1 RAs improve glycemic control, promote cardiovascular and renal protection, and reduce all-cause mortality [2, 3]. Despite this, recent safety concerns have emerged about a potential link to suicidal behavior [4]. Due to 150 spontaneous reports to EMA regarding suicidal ideation [5] EMA and FDA have launched independent investigations into the potential risk of suicide and suicide attempts among users of GLP-1 RAs. However, these investigations remain inconclusive [6]. The mechanism by which GLP-1 RAs potentially increase suicide or suicide attempts is unclear but may involve an overactivation of the hypothalamic-pituitary-adrenal (HPA) axis [7], rapid weight loss [8], and reduced expression of brain-derived neurotrophic factor which may link weight changes to heightened anxiety and suicidal risk [9]. Along this line, a recent large case-control study from 2024 including 162,253 obese patients treated with GLP-1 RAs and a matched group of untreated patients found an association between GLP-1 RAs and increased risk of depression, anxiety and suicidal behavior [10]. In contrast, other large observational cohort studies have not shown any significant associations between treatment with GLP-1 RAs and risk of suicide or suicide attempts, respectively [11, 12]. Similarly, a recent systematic review and meta-analysis including 27 randomized controlled trials (RCT’s) found no association between GLP-1 RAs and increased incidence of suicidal ideation or suicide [13].

It is well established that individuals with mental illness have a higher risk of suicide [14, 15]. A Danish nationwide registry study, including 116,699 individuals with type 2 diabetes and 116,008 matched controls without diabetes, revealed that treatment with GLP-1 RAs was associated with a lower risk of depression [16]. Also, a meta-analysis on antidepressant effects of GLP-1 RA including 2071 participants showed reduced depression scores with GLP-1 treatment compared with controls [17]. This could suggest that GLP-1 may have a protective effect on suicide risk [18]. A systematic review of 26 studies including 3020 participants showed mixed and inconclusive effects of GLP-1 RAs on psychiatric symptoms. Some studies indicated potential benefits, and others found no significant effects. Given that GLP-1 RAs may offer therapeutic options for addressing psychiatric symptoms, it is crucial to evaluate the potential risk of increased suicide rates associated with their use. Thus, we aimed to examine the association, if any, between the use of GLP-1 RAs and the risk of suicide or suicide attempts.

We hypothesized that:

(I) Treatment with GLP-1 RAs would not be associated with a higher risk of suicide than treatment with sodium-glucose cotransporter-2 (SGLT-2) inhibitors and dipeptidyl peptidase-4 (DPP-4) inhibitors.
(II) Suicide risk would be the same or lower after treatment with GLP-1 RAs compared with the period before treatment.

## Material and methods

### Study design and population

We applied a new-user, active comparator cohort study design, comparing GLP-1 RA users with SGLT-2 inhibitor users and GLP-1 RA users with DPP-4 inhibitor users on the incidence of suicide or suicide attempts [19], utilizing information from registers on the entire Danish population [20]. The study population was identified in the Danish Civil Registration system using the unique personal identification number (CPR), ensuring precise linkage of recorded individual-level information across registers [21]. We included two cohorts. In the first cohort we compared GLP-1 RA users with SGLT-2 inhibitor users during the study period 2014-2022. In the second cohort, we compared GLP-1 RA users with DPP-4 inhibitor users from January 2009-2022. The new GLP-1 RA users, SGLT-2 inhibitor users and DPP-4 inhibitor users were defined as a first-time redemption for prescription of drugs with anatomical therapeutic chemical (ATC) codes A10BJ (GLP-1), A10BH (DPP-4 inhibitors) or A10BK (SGLT-2 inhibitors). We selected SGLT-2 inhibitors and DPP-4 inhibitors as active comparators, because they are used at a similar disease stage as GLP-1 and are not associated with the outcome. Furthermore, it enhances the validity and reduces potential bias. During the study period GLP-1 RA were approved for use only in patients with type 2 diabetes; prescribing for overweight or obesity was largely off-label, as this indication was not authorized until December 2022. The Index date was set as the first prescription of GLP-1 RA, SGLT-2 inhibitors or DPP-4 inhibitors. We excluded individuals prescribed with GLP-1 RAs or SGLT-2 inhibitors before index date in the GLP-1 RA vs. SGLT-2-inhibitor-cohort and individuals prescribed with GLP-1 RA or DPP-4 inhibitors prior to index date in the GLP-1 RA vs. DPP-4 inhibitor cohort, individuals who immigrated or died before the index date or age <18 years at first redemption.

We also used a self-controlled case series design [19], which comprised of new users of GLP-1 RAs (*n* = *85,717*) or SGLT-2 inhibitors (*n* = *78,366*) or DPP-4 inhibitors (*n* = 35,762) from 2014 to 2022. We assessed the risk of suicide or suicide attempts following initiation of GLP-1 RA, DPP-4 inhibitors or SGLT-2 inhibitors, respectively. The follow-up time for each individual was divided into three time periods: (1) pretreatment (12 months before initiation), (2) initial exposed period (0-12 months), and (3) late exposed period (13-24 months). This allows distinction between short term effect under up titration and more sustained impacts of GLP-1 RA, DPP-4 inhibitors or SGLT-2 inhibitors. All individuals were unique users and had not received any of the three medications prior.

All prescription refills for medication with ATC codes A10BJ (GLP-1 RAs), A10BH (DPP-4 inhibitors) or A10BK (SGLT-2 inhibitors) were identified in the Danish National Prescription Registry (DNPR) which had recorded all prescribed drugs including date of prescription redemption and ATC codes [22].

Treatment duration was based on the number of daily defined doses filled, with 90 extra follow-up days for irregular use. A gap over 90 days between prescription fills marked the end of treatment, to account for immortal time bias [23].

In Denmark GLP-1 RAs were approved in 2006, DPP-4 inhibitors in 2007, and SGLT-2 inhibitors in 2012. The study start date was set when approximately 3000 received medication, to minimize risk of channeling bias [24].

### Outcomes: Suicide or suicide attempts

Primary outcome was suicide and suicide attempts. Suicide attempts were defined using International Classification of Disease version 10 (ICD-10) codes from the Danish National Patient Register (DNPR): ICD-10 T39, T42, T43, X60-X64, X66-X84. Suicides were identified through the causes-of-death registry using all cases coded as suicides from 2009-2022.

### Covariates

We selected covariates assumed to be associated with selection of patients for GLP-1 RAs, SGLT-2 inhibitors, DPP-4 inhibitors and risk of suicide or suicide attempts from the Danish National Patient Registry and Danish National Prescription Registry. We defined somatic comorbidity as having type 1 and 2 diabetes (ICD-10: E10, E11, E14), obesity (ICD-10: E66), heart disease (ICD-10: I20-I25), stroke (ICD-10: I61, I63, I64, G45) within 10 years prior to index date. We defined psychiatric comorbidity as having alcohol and substance abuse (ICD-10: F10-F19), schizophrenia (ICD-10: F20-F29), bipolar disorders (ICD-10: F30-F31), major depression (ICD-10: F32-F33), eating disorders (ICD-10: F50), personality disorders (ICD-10: F60-F62) and psychotropics within 10 years prior to index date (**See** Supplementary Table 1). We obtained information on sex, age, marital status, and region of residence at the index date from the Danish Civil Registration System. Educational status at index date was obtained from the Population’s education register using the International Standard Classification of Education (ISCED): 0-2 (basic education) 3 + 5 (middle education) and 6-8 (high education) [25]. The self-controlled time series design removes time constant confounders [26], therefore we did not adjust for them in the analysis.

### Statistical analysis

We used cumulative incidences and cause specific hazard regression to estimate hazard ratios (HR) and 95% confidence intervals (CI) for risk of suicide and suicide attempts in GLP-1 RA users vs SGLT-2 inhibitor users or DPP-4 inhibitor users, taking death as a competing risk to suicide into account. In the cohorts we adjusted for age, sex, marital status, education, physical and psychiatric comorbidity registered within 10 years before the index date.

In the intention-to-treat (ITT) analysis, cohort members were followed from index date until first registration of suicide or suicide attempts, emigration, death, switching to the other study drug or end of follow-up (31 December 2022). We also conducted an “as treated” analysis assessing only active treatment from first initiation until first break of treatment, where cohort members were followed from index date until first registration of suicide or suicide attempts, emigration, death, switching to the other study drug or reaching the end of their first treatment period. In sensitivity analyses, we investigated whether recent psychiatric history, defined as any hospitalization in a psychiatric department, psychiatric consultation or dispensing of psychotropics that occurred within the year preceding the index date, altered the association between GLP-1 RA and the risk of suicide or suicide attempts. In the analyses we compared the use of GLP-1 RAs and use of SGLT-2 inhibitors or DPP-4 inhibitors, respectively. The sensitivity analyses were adjusted for age, sex, marital status, education, physical comorbidity.

To assess risk variation over time, we stratified the analyses on 0-1 year and 0-3 years by splitting the data on follow up time. To assess if risk of suicide or suicide attempts vary with sex and age, we explored possible interactions between these and prior suicide attempts using likelihood ratio test. In the self-controlled case series design, we used Poisson regression to estimate suicide or suicide attempts across study periods, calculating incidence rate ratio (IRR) with 95% confidence intervals using the pretreatment period as reference.

## Results

In the GLP-1 RA vs SGLT-2 inhibitor cohort (2014-2022) we identified 83,464 new users of GLP-1 RAs and 78,366 new users of SGLT-2 inhibitors. In the GLP-1 vs DPP-4 inhibitor cohort (2009-2022), we identified 108,322 new users of GLP-1 RAs and 55,411 new users of DPP-4 inhibitors using the National Prescription Registry.

Descriptive data are presented in Table 1. At the index date in GLP-1 RA vs SGLT-2 inhibitor cohort, GLP-1 RA users were generally younger (median age 55 years [25-75 percentile: 45-65] vs 66 years [25-75 percentile: 57-74] more often female (59% vs 36%) and had a higher level of education compared with SGLT-2 inhibitor users (21% vs 15% were within ISCED level 6-8). In the GLP-1 RA vs DPP-4 inhibitor cohort, we observed the same pattern. Additionally, GLP-1 RA users had a higher prevalence of psychiatric comorbidity and more often received psychotropics compared with SGLT-2 and DPP-4 inhibitor users (20% in GLP-1 RA users vs 13% in SGLT-2 inhibitor users and 14% in DPP-4 inhibitor users, respectively).

**Table 1:** Comparison of baseline characteristics between individuals treated with GLP-1 RAs, SGLT-2 inhibitors, or DPP-4 inhibitors.

	Cohort 1		Cohort 2	
	GLP-1 N = 83,464	SGLT-2 N = 78,366	GLP-1 N = 108,322	DPP-4 N = 55,411
**Age (years)**	55 (45, 65)	66 (57, 74)	56 (47, 65)	68 (58, 76)
**Sex (female)**	48,950 (59)	27,854 (36)	59,142 (55)	23,807 (43)
**Married %**	44,669 (54)	43,794 (56)	59,225 (55)	30,059 (54)
**Education level**				
**ISCED 0-2**	23,321 (28)	27,623 (35)	32,357 (30)	23,353 (42)
**ISCED 3 + 5**	41,411 (50)	37,472 (48)	53,415 (49)	24,027 (43)
**ISCED 6–8**	17,775 (21)	11,623 (15)	21,143 (20)	6439 (12)
**Unknown level**	957 (1.1)	1648 (2.1)	1407 (1.3)	1592 (2.9)
**Income level**				
**Low**	24,052 (29)	29,741 (38)	29,525 (27)	24,983 (45)
**Middle**	25,748 (31)	28,062 (36)	34,945 (32)	19,579 (35)
**High**	33,525 (40)	20,267 (26)	43,697 (40)	10,812 (20)
**Unknown**	139	296	155	37
**Region of Denmark**				
**Northern Denmark**	6403 (7.7)	8821 (11)	8256 (7.6)	7443 (13)
**Mid Denmark**	16,659 (20)	17,520 (22)	21,954 (20)	11,916 (22)
**South Denmark**	16,859 (20)	17,238 (22)	21,530 (20)	12,455 (22)
**Capital**	26,780 (32)	21,962 (28)	34,531 (32)	15,373 (28)
**Zealand**	16,763 (20)	12,825 (16)	22,051 (20)	8224 (15)
**Psychiatric diagnosis (the preceding 10 years)**				
**Schizophrenia**	973 (1.2)	756 (1.0)	1205 (1.1)	526 (0.9)
**Major depressive disorder**	2331 (2.8)	1234 (1.6)	2694 (2.5)	905 (1.6)
**Bipolar disorder**	401 (0.5)	210 (0.3)	465 (0.4)	182 (0.3)
**Eating disorder**	197 (0.2)	17 (<0.1)	208 (0.2)	5 (<0.1)
**Personality disorders**	1136 (1.4)	290 (0.4)	1267 (1.2)	190 (0.3)
**Alcohol or substance abuse**	1955 (2.3)	1683 (2.1)	2317 (2.1)	1061 (1.9)
**Psychiatric comorbidity**	6993 (8.4)	4190 (5.3)	8156 (7.5)	2869 (5.2)
**Psychiatric comorbidity with medication**	16,839 (20)	10,413 (13)	20,433 (19)	7792 (14)
**Psychotropic treatment**				
**Antidepressants**	10,949 (13)	6330 (8.1)	13,432 (12)	4859 (8.8)
**Antipsychotics**	1772 (2.1)	1208 (1.5)	2192 (2.0)	1085 (2.0)
**Anticonvulsants**	706 (0.8)	420 (0.5)	821 (0.8)	255 (0.5)
**Lithium**	67 (<0.1)	46 (<0.1)	73 (<0.1)	48 (<0.1)
**Somatic diagnosis (the preceding 10 years)**				
**Heart disease**	4832 (5.8)	11,954 (15)	6996 (6.5)	5345 (9.6)
**Stroke**	1299 (1.6)	2424 (3.1)	1627 (1.5)	1592 (2.9)
**Obesity**	8313 (10.0)	1607 (2.1)	10,764 (9.9)	1783 (3.2)
**Diabetes type 2**	20,636 (25)	25,939 (33)	34,593 (32)	25,692 (46)
**Time since diabetes type 2 onset (years)**	0.4 (0.0, 7.4)	5.2 (0.6, 10.4)	2.4 (0.0, 7.7)	5.7 (2.2, 9.1)
**Receiving Antidiabetics**	67,262 (81)	69,946 (89)	88,296 (82)	48,625 (88)
**Time with antidiabetic medication (years)**	0.4 (0.0, 7.1)	4.9 (0.5, 10.1)	2.2 (0.0, 7.2)	5.4 (2.0, 8.7)
**Suicide or suicide attempts**				
**Suicide or suicide attempts before index**	2511 (3.0)	1354 (1.7)	2985 (2.8)	924 (1.7)
**Suicide or suicide attempts after index**	118 (0.1)	117 (0.1)	283 (0.3)	224 (0.4)
**Suicide after index**	9 (<0.1)	22 (<0.1)	35 (<0.1)	38 (<0.1)
**Suicide attempts after index**	111 (0.1)	97 (0.1)	256 (0.2)	189 (0.3)

Categorical variables are presented as n (%).

*ISCED*, International Standard Classification of Education; *GLP-1*, glucagon-like peptide-1 receptor agonist; *SGLT-2*, sodium-glucose cotransporter-2 inhibitors; *DPP-4*, dipeptidyl peptidase-4 inhibitors.

### Association of GLP-1 RAs with suicide or suicide attempts

In intention-to-treat (ITT) analysis the GLP-1 RA vs SGLT-2 inhibitor cohort median follow-up time was 1 year [1–3] for new GLP-1 RA users and 2 years [1–3] for new SGLT-2 inhibitor users. In as-treated analyses median follow-up was 1 year [0-2] for both new GLP-1 RA users and new SGLT-2 inhibitor users.

In ITT analysis the GLP-1 RA vs DPP-4 inhibitor cohort median follow-up was 2 years [1–4] for GLP-1 RAs users and 4 years [2–7] for DPP-4 inhibitor users. In the as-treated analysis median follow-up was 1 year [0-2] for GLP-1 RA users and 1 year [1–3] for users of DPP-4 inhibitors.

In the unadjusted cause-specific-hazard regression model there was no difference in the risk of suicide or suicide attempts between patients treated with GLP-1 RAs compared with patients treated with SGLT-2 inhibitors or DPP-4 inhibitors, respectively, after 1 year and 3 years of follow-up, see Table 2.

**Table 2:** The association between use of GLP-1 RAs and suicide or suicide attempts compared with use of SGLT-2 inhibitors or DPP-4 inhibitors in Danish adults.

	GLP-1/SGLT-2	GLP-1/SGLT-2	GLP-1/DPP-4	GLP-1/DPP-4
**Follow-up time (years)**	0–1	0–3	0–1	0–3
**Intention-to-treat analysis**				
**Number of suicide or suicide attempts**	43/34	92/95	40/52	112/155
**Unadjusted Hazard Ratio**	1.27 [0.81;2.00]	1.01 [0.76;1.35]	0.79 [0.52;1.19]	1.00 [0.78;1.28]
Adjusted * Hazard Ratio	0.93 [0.57;1.52]	0.83 [0.61;1.13]	0.58 [0.37;0.91]	0.81 [0.63;1.06]
**As-treated analysis**				
**Number of suicide or suicide attempts**	35/29	62/60	35/39	64/84
**Unadjusted Hazard Ratio**	1.27 [0.78;2.08]	1.12 [0.77;1.63]	0.74 [0.47;1.18]	1.02 [0.73;1.41]
Adjusted * Hazard Ratio	0.92 [0.55;1.55]	1.00 [0.70;1.43]	0.56 [0.34;0.92]	0.84 [0.60;1.19]

*GLP-1*, glucagon-like peptide-1 receptor agonists; *SGLT-2*, sodium-glucose cotransporter-2 inhibitors; *DPP-4*, dipeptidyl peptidase-4 inhibitors; CI: 95% confidence interval.

*Adjusted analyses were adjusted for age, sex, marital status, education, physical and psychiatric comorbidity.

In the GLP-1 RA vs SGLT-2 inhibitor cohort, there was no difference in the adjusted HR of suicide or suicide attempts between patients treated with GLP-1 RAs compared with patients treated with SGLT-2 inhibitors after 1 year of follow-up (HR_365_ 0.93 [0.57;1.52]), see Table 2. In the adjusted cause-specific-hazard regression for the GLP-1 RA vs DPP-4 inhibitor cohort, GLP-1 RAs were associated with a lower risk of suicide or suicide attempts compared with DPP-4 inhibitors after 1 year follow-up (HR_365_ 0.58 [0.37;0.91]). Results were similar across both cohorts in the as-treated analyses, Table 2.

In sensitivity analyses, see Table 3, GLP-1 RA use was associated with lower risk compared with SGLT-2 inhibitors among patients without a recent psychiatric history, but no difference was observed among patients with recent psychiatric history. In the GLP-1 RA vs DPP-4 inhibitor cohort, GLP-1 RA use was associated with lower risk among patients with recent psychiatric history, and no difference among patient without recent psychiatric history.

**Table 3:** The association between use of GLP-1 RAs and suicide or suicide attempts compared with use of SGLT-2 inhibitors or DPP-4 inhibitors with or without recent psychiatric history in Danish adults.

	GLP-1/SGLT-2	GLP-1/SGLT-2	GLP-1/DPP-4	GLP-1/DPP-4
**Follow-up time (years)**	0–1	0–3	0–1	0–3
**Intention-to-treat analysis**				
**Hazard Ratio without psychiatric history**	0.58 [0.21;1.60]	0.64 [0.37;1.08]	0.55 [0.21;1.46]	0.87 [0.55;1.40]
**Hazard Ratio with psychiatric history**	0.97 [0.55;1.71]	0.88 [0.60;1.28]	0.52 [0.31;0.86]	0.71 [0.52;0.97]
**As-treated analysis**				
**Hazard Ratio without psychiatric history**	0.57 [0.20;1.64]	0.48 [0.25;0.93]	0.51 [0.18;1.50]	0.78 [0.41;1.48]
**Hazard Ratio with psychiatric history**	1.00 [0.54;1.84]	1.06 [0.67;1.68]	0.51 [0.29;0.89]	0.80 [0.53;1.21]

Analyses were adjusted for age, sex, marital status, education, physical comorbidity and were stratified by recent psychiatric history.

*GLP-1*, glucagon-like peptide-1 receptor agonists; *SGLT-2*, sodium-glucose cotransporter-2 inhibitors; *DPP-4*, dipeptidyl peptidase-4 inhibitors; *CI*, 95% confidence interval.

We found no interactions, except between prior suicide attempts and sex in the comparison of GLP-1 RAs and SGLT-2 inhibitors, indicating that the effect of prior suicide attempts on the risk of a subsequent suicide attempt is greater among men.

**Fig. 1:**
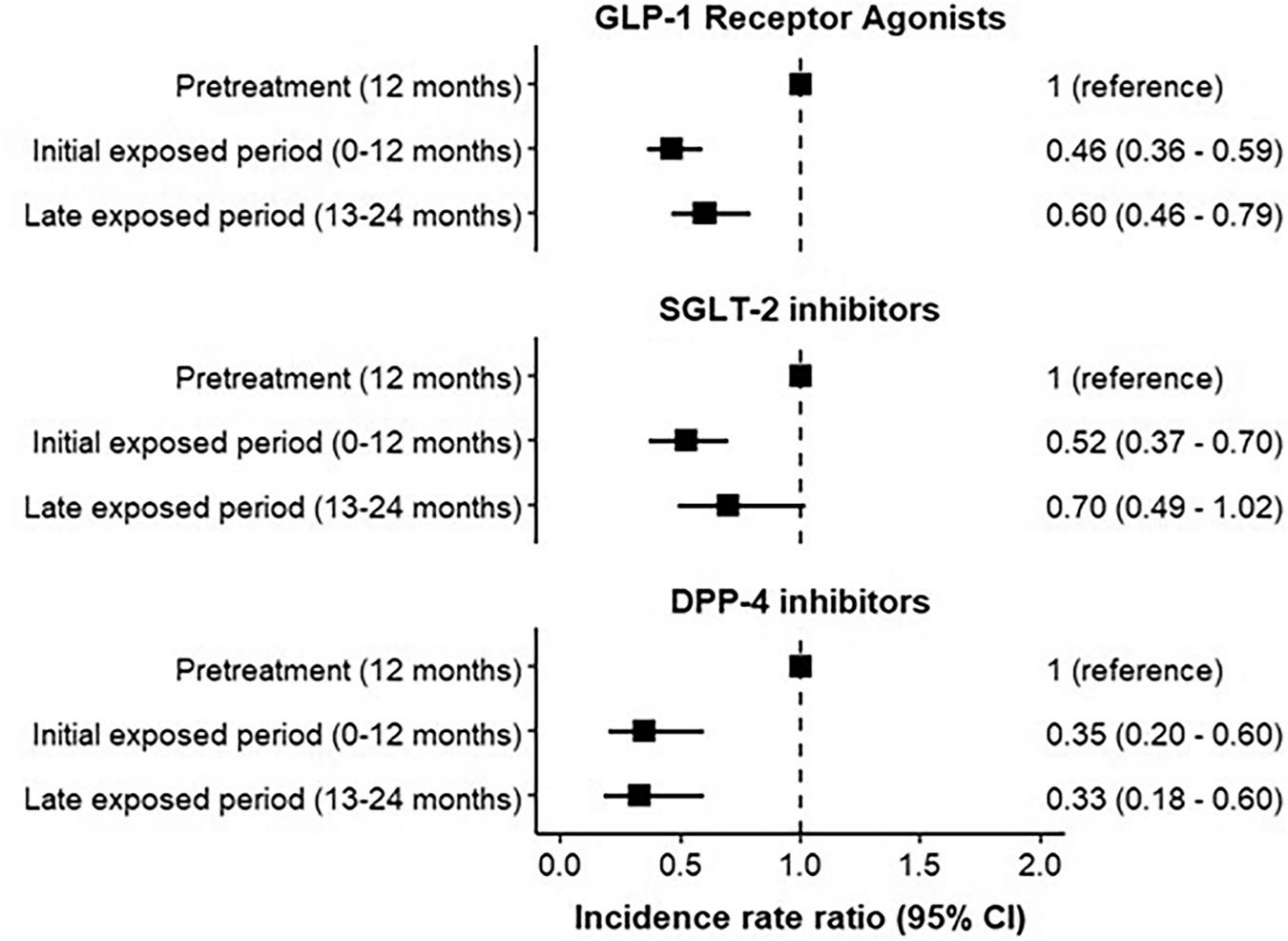
Incidence rate ratios of suicide attempts or suicide across three predefined time periods in patients who initiate treatment with GLP-1 RAs (n = 85,717), SGLT-2 inhibitors (n = 78,366) or DPP-4 inhibitors (n = 35,762). GLP-1 glucagon-like peptide-1 receptor agonist, SGLT-2 sodium-glucose cotransporter-2 inhibitors, DPP-4 dipeptidyl peptidase-4 inhibitors.

In the self-controlled analysis, we compared incidence rates in periods before and after initiation of GLP-1 RAs within individuals. The risk of suicide or suicide attempts was lower during both the initial exposed period (0-12 months) and late exposed period (13-24 months) compared to the pretreatment period (Fig. 1). Similar patterns were observed for SGLT-2 inhibitors during the initial exposed period (0-12 months) and the late exposed period (13-24 months). For DPP-4 inhibitors the IRRs were likewise lower in both periods, compared to the pretreatment period (Fig. 1).

## Discussion

In this nationwide register study, we investigated the association between new users of GLP-1 RAs, SGLT-2 inhibitor users and DPP-4 inhibitor users and the risk of suicide or suicide attempts. Overall, GLP-1 RA treatment did not increase the incidence of suicide or suicide attempts in either the active comparator new-user design or in the self-controlled case series design.

### Comparison with previous studies

Our findings align with prior studies reporting no association between GLP-1 RAs and suicide or suicide attempts. A meta-analysis of 27 randomized clinical trials [13] found no association between GLP-1 RAs and suicidal ideation or suicide. However, the included trials were conducted on relatively small sample sizes, which may limit the ability to detect rare psychiatric outcomes. Along this line, a recent meta-analysis of 11 heterogeneous observational studies primarily consisting of pharmacovigilance and cohort studies reported no significant difference in suicidal outcomes between GLP-1 RAs compared with other anti-hyperglycemic drugs (RR:0.568, 95% Cl:0.077-4.205) [27].

Consistent with our findings, four cohort studies found no increased risk of suicidal ideation between GLP-1 RAs and SGLT-2 inhibitors [11, 12, 28, 29]. One of these, a U.S. national Medicare cohort study, also included 21,402 pairs of patients treated with a GLP-1 RA versus DPP-4 inhibitors and found no differences in suicidal ideation and behaviors between groups [29]. One study revealed a possible protective association between GLP-1 RA and reduced risk of suicidal behavior (HR: 0.27, 95% Cl 0.20 to 0.36) [12]. However, a large Taiwanese cohort study of 11.7 million obese individuals found that GLP-1 RAs, particularly semaglutide, were associated with increased risk of depression, anxiety, and suicidal behavior [10]. Many of the previous studies had methodological limitations such as selection bias [10, 12], and limited generalizability [11, 29].

We used GLP-1 RA users, SGLT-2 inhibitor users and DPP-4 inhibitor users in the cohort analysis as we assumed that these individuals would be similar. However, differences in sociodemographic profile and comorbidities were present, such as higher incomes and educational level of GLP-1 RAs users compared with SGLT-2 inhibitor users and DPP-4 inhibitor users suggesting selection bias in prescription. Despite a higher prevalence of psychiatric comorbidity GLP-1 RA users showed no increased risk of suicide compared with SGLT-2 inhibitor users and DPP-4 inhibitor users in our analysis. Despite GLP-1 RA users did not exhibit increased risk of suicide, they had a higher prevalence of psychiatric comorbidity. This can possibly reflect underlying mental pain - subjective psychological distress that is not directly captured in register-based data, but remains clinically relevant for understanding psychiatric risk profiles [30].

In our self-controlled design, no evidence of increased risk of suicide or suicide attempts following GLP-1 RA initiation was observed, consistent with a French case-time-control study including 1102 patients with suicide attempt or fatal suicide and 5,494 controls from 2013-2021 [31]. In fact, lower IRRs were observed during both the initial (0–12 months) and late (13–24 months) exposed periods compared with pretreatment, a pattern also seen for SGLT-2 and DPP-4 inhibitors, possibly reflecting increased healthcare contact or a healthier lifestyle during treatment. Treatment initiation may be accompanied by closer clinical follow-up, lifestyle advice and increased patient engagement, which may influence suicide risk independent of GLP-1 RA effects.

In sum, most studies suggest that GLP-1 RAs do not increase the risk of suicide, with some indicating potential benefits; nevertheless, a few studies have reported an increased risk of suicide or suicide attempts. Our large-scale nationwide study shows that GLP-1 RAs do not seem linked to a higher risk of suicide or suicide attempts.

### Strengths and limitations

Our findings are robust, based on two large population-based cohorts using comprehensive nationwide registers in Denmark. Free healthcare enhances generalizability, and unique personal identifiers enabled complete linkage across registers, providing full follow-up on suicide and suicide attempts from mortality and hospital data. Using both SGLT-2 and DPP-4 inhibitors as active comparators strengthened study validity, while register linkage minimized selection and recall bias by ensuring exposures were recorded independently of outcomes.

First, lack of exposure is possible, as we lacked information on patient compliance with prescribed treatments and potential lack of medication dispensed at hospitals, however as medication compliance is similar across all three groups and we defined exposure as redeemed prescriptions, ensuring that the medication was actively collected by the patient, systematic misclassification is unlikely. Conversely, although off-label use of GLP-1 RA for overweight and obesity cannot be excluded, such use was likely limited in the present study given existing prescription guidelines and regulatory vigilance.

Second, misclassification of the cause of death is a potential concern, however, deaths from unnatural causes of death are commonly subject to autopsy and the validity of suicide is known to be high in Denmark [32].

Third, the self-controlled case series design accounts for time-invariant confounders like genetic predisposition and personality traits. However, time-varying factors, such as age, may still influence the association. In the context of risk of confounding by indication, propensity score matching was not expected to provide additional reduction of residual confounding beyond the applied covariate adjustment (psychiatric, somatic, and sociodemographic factors) and self-controlled analyses [33]. Regarding potential confounding by indication related to an obesity diagnosis, the number of outcomes in individuals diagnosed with obesity according to the registers is too low to carry out a meaningful subgroup analysis. This is due to underreporting of overweight and obesity diagnoses in the Danish registries, and the fact that GLP-1 receptor agonists were not approved for the treatment of obesity until December 2022.

Fourth, the competing risk of death could underestimate suicide risk. As this issue also applies to previous studies, the overall evidence may underestimate risk. Nonetheless, we replicated prior findings, supporting that use of GLP-1 RA was not associated with an increased suicide risk. These findings are most comparable to populations with metabolic disease in healthcare systems with similar access and registration of suicides.

Taken together, the observed lack of association between the use of GLP-1 RA and suicide or suicide attempts reflects the absence of increased suicide risk, even when accounting for the acknowledged limitations. The methodological robustness of the study, and consistency with recent literature support that use of GLP-1 RA does not appear to be associated with an increased risk of suicide or suicide attempts.

Future work is recommended to pool multinational datasets to increase precision of estimates, given the low number of suicide and suicide attempt outcomes in the study population.

## Conclusion

This extensive nationwide registry study found no evidence of a higher risk of suicide following GLP-1 RA initiation. Overall, the use of GLP-1 RAs was not associated with a higher incidence of suicide or suicide attempts in new-user active comparator cohort analyses or in self-controlled case series analyses.

## Supplementary information

Supplementary Table 1

## Data Availability

The data that support the findings of this study are available from Statistics Denmark. Restrictions apply to the availability of these data, which were used under license for this study.
