# Supplementary material for: GLP-1 receptor agonists and risk of suicide or suicide attempts – A nationwide cohort and self-controlled case series study
Source: Mol Psychiatry. 2026 Apr 18;31(9):5316–22. doi: 10.1038/s41380-026-03619-y (PMC13441866; doi:10.1038/s41380-026-03619-y)
Supplement: Supplementary file 1 [file 41380_2026_3619_MOESM1_ESM.pdf]

Supplementary Table 1. Codes used for definition of comorbidity

Baseline comorbidity	ICD-10 codes	ATC codes
Comorbid heart disease	I20-I25	
Comorbid stroke	I61, I63, I64, G45	
Comorbid diabetes	E10, E11, E14	
Prior antidiabetic treatment		A10A, A10B
Antidepressant medication		N06A
Antipsychotic medication		N05A
Anticonvulsants		N03AG01, NO3AX09
Lithium		N05AN
Comorbid obesity	E66	
Alcohol and drug misuse	F10-F19	N07BB, N05BA2
Schizophrenia	F20-F29	
Major depression	F32-F33	
Bipolar disorder	F30-F31	
Eating disorder	F50	
Personality disorders	F60-62	
